# Evaluation of noscapine-licorice combination effects on cough relieving in COVID-19 outpatients: A randomized controlled trial

**DOI:** 10.3389/fphar.2023.1102940

**Published:** 2023-02-17

**Authors:** Saghar Barati, Faezeh Feizabadi, Hakimeh Khalaj, Hakimeh Sheikhzadeh, Hamid R. Jamaati, Hirad Farajidavar, Farzaneh Dastan

**Affiliations:** ^1^ Department of Clinical Pharmacy, Shahid Beheshti University of Medical Sciences, Tehran, Alborz, Iran; ^2^ National Research Institute of Tuberculosis and Lung Diseases, Shahid Beheshti University of Medical Sciences, Tehran, Alborz, Iran; ^3^ Chronic Respiratory Diseases Research Center, National Research Institute of Tuberculosis and Lung Diseases, Tehran, Alborz, Iran

**Keywords:** COVID-19, cough, glycyrrhiza, noscapine, outpatients

## Abstract

**Background:** As February 2023, SARS-CoV-2 is still infecting people and children worldwide. Cough and dyspnea are annoying symptoms almost present in a large proportion of COVID-19 outpatients, and the duration of these symptoms might be long enough to affect the patients’ quality of life. Studies have shown positive effects for noscapine plus licorice in the previous COVID-19 trials. This study aimed to assess the effects of the combination of noscapine and licorice-for relieving cough in outpatients with COVID-19.

**Methods:** This randomized controlled trial was conducted on 124 patients at the Dr. Masih Daneshvari Hospital. Participants over 18 years of age with confirmed COVID-19 and cough were allowed to enter the study if the onset of symptoms was less than 5 days. The primary outcome was to assess the response to treatment over 5 days using the visual analogue scale. Secondary outcomes included the assessment of cough severity after 5 days using Cough Symptom Score, as well as the cough-related quality of life and dyspnea relieving. Patients in the noscapine plus licorice group received Noscough^®^ syrup 20 mL every 6 h for 5 days. The control group received diphenhydramine elixir 7 mL every 8 h.

**Results:** By day five, 53 (85.48%) patients in the Noscough^®^ group and 49 (79.03%) patients in the diphenhydramine group had response to treatment. This difference was not statistically significant (*p*-value = 0.34). The presence of dyspnea was significantly lower in the Noscough^®^ group *versus* diphenhydramine at day five (1.61% in the Noscough^®^ group vs. 12.9% in the diphenhydramine group; *p*-value = 0.03). The cough-related quality of life and severity also significantly favored Noscough^®^ syrup (*p*-values <0.001).

**Conclusion:** Noscapine plus licorice syrup was slightly superior to diphenhydramine in relieving cough symptoms and dyspnea in the COVID-19 outpatients. The severity of cough and cough-related quality of life were also significantly better in the noscapine plus licorice syrup. Noscapine plus licorice may be a valuable treatment in relieving cough in COVID-19 outpatients.

## Introduction

As of December 2022, seroprevalence studies suggest that up to 80%–90% of the global population has already had an infection with SARS-CoV-2 (Huang et al., 2020). The key strategies to battle the infection are crucial to reducing disease spread—including physical distancing, wearing masks, and avoiding crowds (Dhand and Li, 2020). The cough is a key symptom of COVID-19 comparable to the more common but less severe respiratory infections, including common cold or flu in the acute and post-infective phases of the infection. Moreover, cough increases the risk of community transmission by respiratory droplets, distressing patients and leading to social isolation (Song et al., 2021). Many mechanisms have been suggested for COVID-19- induced cough including neuroinflammation or neuroimmunomodulation *via* the sensory nerves (Song et al., 2021). Another potential mechanism of cough in COVID-19 is bradykinin syndrome which is defined as the reduced degradation of bradykinin in the body that may further lead to dry cough not responding to regular treatments (Alkotaji and Al-Zidan, 2021). Identifying ways to relieve COVID-19-associated cough could help to prevent community transmission and disease spread, along with removing the stigma of this symptom (Dhand and Li, 2020). As well, the exact mechanisms of COVID-19-associated cough are unclear. Accordingly, evidence-based treatment options for COVID-19 cough are needed (Song et al., 2021). The current antitussive medication used for the treatment of cough includes diphenhydramine which has much anticholinergic activity leading to blurred vision, constipation, urinary retention and xerostomia. Most elderly patients will not tolerate these adverse events. Moreover, opioid antitussives including dextromethorphan may induce central nervous system (CNS) depression and they also may be associated with respiratory issues (Enna and Bylund 2008).

Different measures have existed in viral respiratory infections to treat cough. Among them, natural and herbal products are common (Shergis et al., 2015). Products containing noscapine or licorice are used widely to relieve cough (Shergis et al., 2015; Kuang et al., 2018). Noscapine is a naturally occurring opium-isoquinoline alkaloid that is related to papaverine. It acts centrally as a cough suppressant and has actions and uses similar to dextromethorphan (Enna and Bylund 2008). Unlike opioid antitussives, respiratory and CNS depression, as well as addiction, have not been reported with noscapine (Enna and Bylund 2008). Moreover, noscapine can help decrease bradykinin-mediated cytokine release due to Angiotensin converting 2 enzyme inhibition by SARS-CoV-2. In turn, it can reduce tissue damage, especially in the lungs (Ebrahimi, 2020). As the previous studies have shown that the accumulation of bradykinin along with cytokine storms may be the culprit for the pathogenesis of SARS-CoV-2 (Wilczynski et al., 2021). A cytokine storm is a hyperinflammatory state that can lead to excessive production of cytokines by a deregulated immune system (Zanza et al., 2022).

The licorice is a popular traditional Chinese medicine (TCM) used to treat respiratory diseases, including cough, sore throat, asthma, and bronchitis (Kuang et al., 2018).

Noscough^®^ is a natural syrup (containing noscapine and licorice extract) used as an antitussive medication. Based on these reasons, the use of Noscough^®^ may be a safe and effective option for the treatment of COVID-19 cough. Hence, this study aimed to investigate the effects of Noscough^®^ syrup in relieving cough in the outpatients with COVID-19.

## Materials and methods

### Study design

This randomized controlled trial was conducted at Dr. Masih Daneshvari Hospital—a tertiary referral center in Tehran, Iran, affiliated with Shahid Beheshti University of Medical Sciences (SBMU).

The study was approved by the Ethics Committee of SBMU, Tehran, Iran (Ethics code: IR.SBMU.PHARMACY.REC.1400.252) with registry code of IRCT20151227025726N31 in the Iranian registry of clinical trials (IRCT). Informed written consent was obtained from all patients before allocation.

### Participants

Patients over 18 years of age with cough and positive reverse transcriptase polymerase chain reaction (RT-PCR) test for COVID-19 with the onset time of less than 5 days were included in the study.

Exclusion criteria were as follows: pregnancy or breastfeeding, history of allergy to noscapine, licorice, diphenhydramine, morphine or other excipients of the study medications, history of seizure, diarrhea or diabetes, consumption of warfarin, benzodiazepines, opioid agonists, and other antitussive medications.

### Randomization and blinding

The block balanced randomization method (twenty-five blocks, including four patients in each block) was used to allocate patients to the Noscough^®^ and control groups. In each block, two patients were assigned to the Noscough^®^ group and two to the control group. The participants were not blinded to the study due to the differences in administration schedules.

### Outcomes

The primary outcome of the study was to assess the response to treatment during 5 days. The visual analogue scale (VAS) score was assessed by patients to evaluate treatment response. The VAS employs a linear scoring method with a straight line with calibration of 0, 1, 2–10 cm (scale lines marked from 0 to 100 mm can also be used); 0 indicates asymptomatic, and 10 represents the most serious (Spinou and Birring, 2014). Treatment response was defined as a decrease of ≥50% in the average VAS score.

Secondary outcomes included the assessment of cough severity after 5 days using cough symptom score (CSS). The CSS is a two-part questionnaire referring to symptoms during the day and night. Based on the frequency, intensity, and influence of cough on daily activities and sleep, cough symptoms are scored from 0 to 5, with 0 indicating no cough and five indicating the most severe cough (Wang et al., 2019). VAS and CSS scores were measured at baseline and day five after treatment.

Another secondary outcome included the assessment of the cough-related quality of life *via* a cough-specific quality of life questionnaire (CQLQ). The CQLQ comprises 28 questions regarding cough and its effects on life. This questionnaire is scored with a 4-point Likert scale, with lower scores indicating less impact of cough on health-related quality of life. The CQLQ total score can range from 28 to 112 (Lechtzin et al., 2013). The CQLQ questionnaire was measured and recorded on day one (baseline) and 5 days after treatment.

### Intervention

The patients in the Noscough^®^ group received Noscough^®^ syrup (Faran Shimi, Iran, each 5 mL contain 7 mg noscapine and 5 mg licorice extract), 20 mL every 6 h for 5 days. The control group received diphenhydramine (Pursina, Iran, each 5 mL contain 12.5 mg diphenhydramine) 7 mL every 8 h. No other medications were received. Patients in both groups received cetirizine 10 mg once daily for the relief of coryzal symptoms. Patients were allowed to leave the study at any time. Their demographic characteristics, underlying diseases, and medication histories were recorded at baseline.

### Statistical analysis

The sample size was calculated based on assuming 55% response to treatment for the diphenhydramine group and 80% response for the Noscough^®^ syrup. This difference was estimated based on the investigators’ opinion and evidence-based useful theoretical mechanisms for the antitussive effects of Noscough^®^. Considering 80% power, error type 1 of 0.05 and a 12% drop-out rate, 62 patients were calculated in each group.

The statistical analyses were performed using SPSS software for Windows (Version 23.0; SPSS Inc., Chicago, IL, United States) and STATA 17. Categorical and nominal variables were expressed as frequency (%) and were compared using the Chi-Square test. The risk difference was calculated as a proper effect size for the primary outcome. Continuous variables were expressed as means ± standard deviations or 95% confidence intervals. An ANCOVA model was employed to assess the differences between the patient-reported outcomes using the baseline values as covariates. *p*-values <0.05 were considered significant.

## Results

### Study participants

**FIGURE 1:**
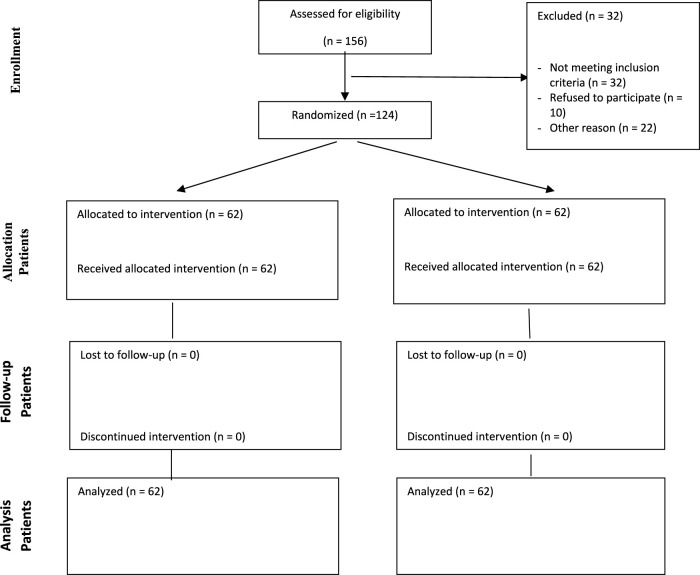
CONSORT diagram showing screening, randomization, and analysis of the participants. The whole participants were included in the analysis.

In total, 124 patients were randomized to diphenhydramine and Noscough^®^ groups equally. The screening and randomization process of the patients are provided in the CONSORT diagram in Figure 1.

Table 1 shows the demographics and past medical histories of the patients. It also demonstrates the baseline values of the patient reported outcomes. There were no meaningful differences regarding the baseline parameters among the two groups.

**TABLE 1:** Demographics and past medical histories of the patients.

Characteristics	Diphenhydramine (*N* = 62)	Noscough^®^ (*N* = 62)
Sex — n (%)		
Male	38 (61.29)	34 (54.83)
Age (y) — mean ± SD	43.88 ± 11.17	44.98 ± 15.3
Medical and drug history — n (%)		
Receiving corticosteroids	36 (58.06)	37 (59.67)
Receiving Remdesivir	36 (58.06)	36 (58.06)
Hypertension	8 (12.09)	9 (14.5)
Diabetes mellitus	7 (11.29)	8 (12.09)
Lung Diseases	0 (0)	4 (6.45)
Chronic kidney diseases	4 (6.45)	1 (1.61)
Immunodeficient	3 (4.83)	2 (3.22)
Baseline cough related characteristics		
CSS — mean ± SD	3.06 ± 1.09	3.2 ± 1.04
VAS — mean ± SD	5.53 ± 2.4	5.98 ± 2.45
CQLQ — mean ± SD	60.95 ± 9.06	63.09 ± 10.35
Dyspnea symptom— n (%)	29 (46.77%)	24 (38.7%)

CSS, cough symptom score; VAS, visual analogue scale; CQLQ, cough-specific quality of life questionnaire.

The results of the primary and secondary outcomes are provided in Table 2. By day five, the incidence of response to treatment in the Noscough^®^ group was 85.48% *versus* 79.03% in the diphenhydramine group. However, this difference was not statistically significant (*p*-value = 0.34). The effect size of this difference was calculated to be 0.06.

**TABLE 2:** Results of the primary and secondary outcomes.

Characteristics	Diphenhydramin (*N* = 62)	Noscough^®^ (*N* = 62)	*p*-value
Patients with response to treatment during 5 days — n (%) ^§^	49 (79.03%)	53 (85.48%)	0.34
Dyspnea at day 5	8 (12.9%)	1 (1.61%)	0.03
CSS day 5 — mean (95% CI) ^¥^	1.53 (1.31—1.76)	1.25 (1.02—1.47)	<0.001
VAS day 5 — mean (95% CI) ^¥^	2.1 (1.69—2.5)	1.42 (1.02—1.83)	<0.001
CQLQ day 5 — mean (95% CI) ^¥^	43.6 (41.38—45.81)	39.91 (37.69—42.12)	<0.001

§: based on the chi-square test.

¥: adjusted based on baseline values (ANCOVA, model).

As the table shows, by day five, dyspnea was still present in 12.9% of the patients in the diphenhydramine group. Whereas only 1.61% of the patients in the Noscugh^®^ group still had dyspnea by the end of the treatment period (*p*-value = 0.03).

As Table 2 shows, considering the baseline values, the results of the quality of life and cough severity were significantly in favor of the Noscough^®^ group (*p*-values <0.001).

## Discussion

The results of our trial confirmed that Noscough^®^ syrup which consists of noscapine and licorice has similar effects with diphenhydramine in terms of response to treatment. However, Noscough^®^ syrup was superior to diphenhydramine in terms of quality of life, dyspnea relief, and cough severity.

Notably, the effect size of the response to treatment as the primary outcome of the study was 0.06 in favor of Noscough^®^ syrup, which is considered a small effect size (Rahlfs and Zimmermann, 2019). Hence, it can be concluded that noscapine may be slightly superior to diphenhydramine in terms of response to treatment, and the study was underpowered to detect this small difference. Moreover, the mean scores of CSS, VAS, and CQLQ, were all in favor of the Noscough^®^ group with significant results with adjustment of their baseline values. The presence of dyspnea was also significantly lower in the Noscough^®^ group by the end of day five. The number needed to treat (NNT) was calculated to be 17, which is also considered a small effect.

Noscough^®^ syrup consists of noscapine and licorice. Noscapine is an opioid antitussive that has been shown to reduce bradykinin-induced cough in humans (Ebrahimi, 2020). Moreover, studies have shown many symptoms related to COVID-19 can be justified with the development of bradykinin (Garvin et al., 2020). Hence, bradykinin and cytokine storms might be associated with worse outcomes in COVID-19. Therefore, mediating these pathways may lead to better symptom-relieving (Rex et al., 2022). Noscapine is considered a safe candidate with the potential benefits of modulating both pathways (Luo et al., 2020; Kumar et al., 2022). The advantages of this medication to diphenhydramine are lack of sedative effects and unlike diphenhydramine it has no anticholinergic effects which is an important advantage in the elderly. In Addition, unlike other opioids, no addiction or respiratory issues have been reported with this medication. Noscugh^®^ side effects only include potential sedative effects in non-pharmacologic doses based on the manufacturer label.

Licorice also has been shown to have antiviral effects against various families, including SARS coronaviruses (Diomede et al., 2021). Moreover, a study using bioinformatics analysis and molecular dynamic stimulation has shown that phaseol in licorice may have beneficial effects in reducing the inflammatory response to COVID-19 by inhibiting the activation of CXCL8 and IL2RA (Cao et al., 2022). An *in silico* analysis performed by Neeraj kumar et al., showed that the combination of noscapine and hydroxychloroquine conjugates has much binding affinity for main protease of SARS-CoV-2 which has critical role in pathogenesis of COVID-19 (Kumar et al., 2022). Other potential effects against COVID-19 symptoms have also been shown for other ingredients of licorice, including Glycerol and Glyasperin F (Cao et al., 2022). Another *in vitro* study has revealed that licorice may block SARS-CoV-2 replication by inhibiting the viral main protease (van de Sand et al., 2021).

The main limitation of the study was that the patients were not blinded to the study interventions. Due to the differences in dosing and interval of administrations blinding was not feasible. Hence, their own believes of the antitussive medication might have influenced the study results. Another limitation is that the investigators had considered a significant effect for Noscough^®^ syrup based on the potential literature-based mechanisms while powering the study. Due to this fact, the study did not meet its primary endpoint as the sample size was not large enough to detect smaller effects. It is suggested to perform the study with larger sample size and also with different reference products as control group including the other opioids.

## Conclusion

Noscough^®^ syrup was slightly superior to diphenhydramine in relieving the symptoms of cough and dyspnea in COVID-19 outpatients. The severity of cough and cough-related quality of life were also in favor of Noscough^®^ syrup significantly. Considering the favorable safety profile of this syrup, Noscough^®^ may be a valuable treatment in relieving cough in COVID-19 outpatients.

## Data Availability

The raw data supporting the conclusion of this article will be made available by the authors, without undue reservation.
